# Supplementary material for: Improving Water Stability of Soil Aggregates with Polyvinyl Alcohol as a Polymeric Binder
Source: Polymers (Basel). 2024 Jun 21;16(13):1758. doi: 10.3390/polym16131758 (PMC11243888; doi:10.3390/polym16131758)
Supplement: Supplementary file 1 [file polymers-16-01758-s001.zip › polymers-3000137-supplementary.pdf]

Supporting Information

Improving Water Stability of Soil Aggregates with Polyvinyl Alcohol as a Polymeric Binder

Chunyan Cao [1], Minkun Cai [2], Lingyu Zhao [2,*] and Gang Li [2,*]

[1] School of Electrics and Computer Engineering, Nanfang College, Guangzhou, Guangzhou 510970, China; caochy@nfu.edu.cn
[2] Department of Materials Science and Engineering, Southern University of Science and Technology, Shenzhen 518055, China; 11930496@mail.sustech.edu.cn
* Correspondence: zhaoly@sustech.edu.cn (L.Z.); 11930690@mail.sustech.edu.cn (G.L.)

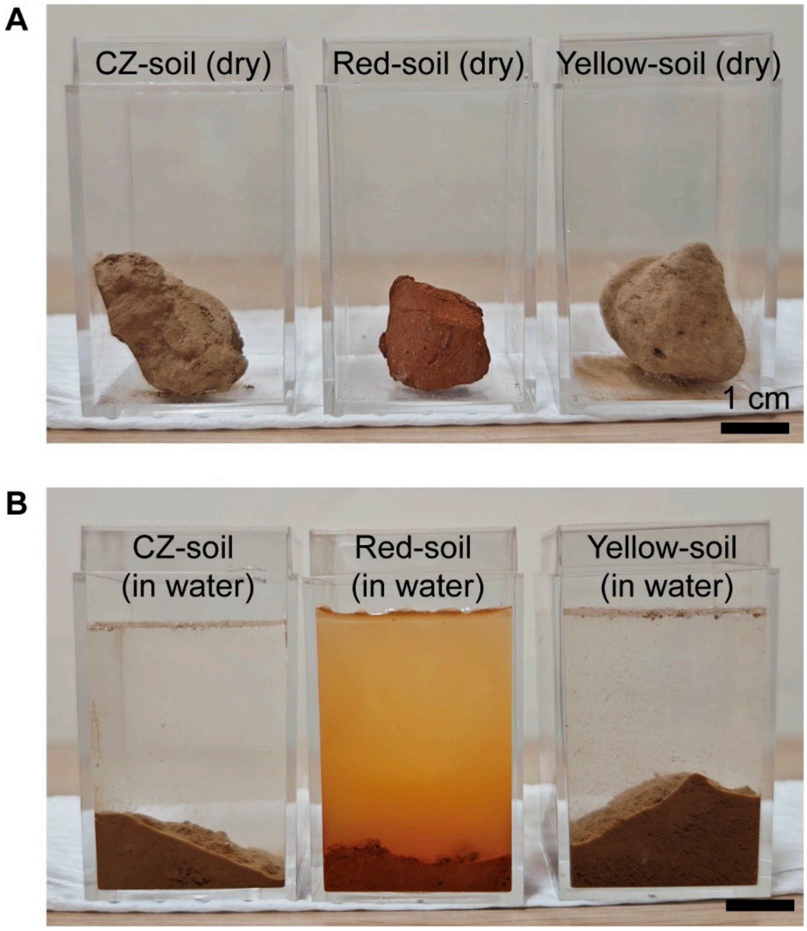

Figure S1. Soil disintegration in water. The large grain of soil (A) breaks down into water and loses its initial shape (B).

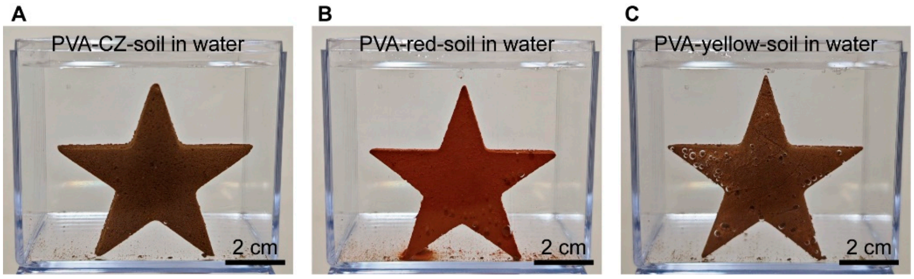

Figure S2. PVA-soil in water. The PVA-CZ soil (A), PVA-red-soil (B), and PVA-yellow-soil, with a star shape, show integrity in water, demonstrating their remarkable stability in water.

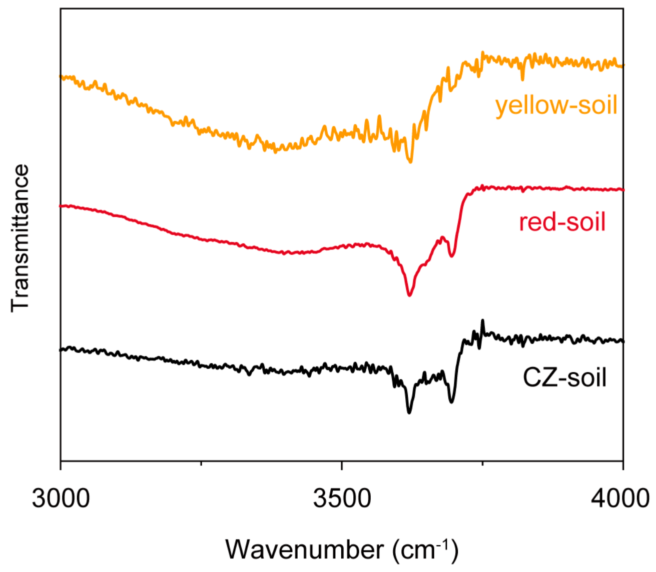

Figure S3. TIR-ATR spectra of yellow soil, red soil, and CZ soil reveal the presence of O-H stretching in the range from 3500-3700 cm^{-1}.

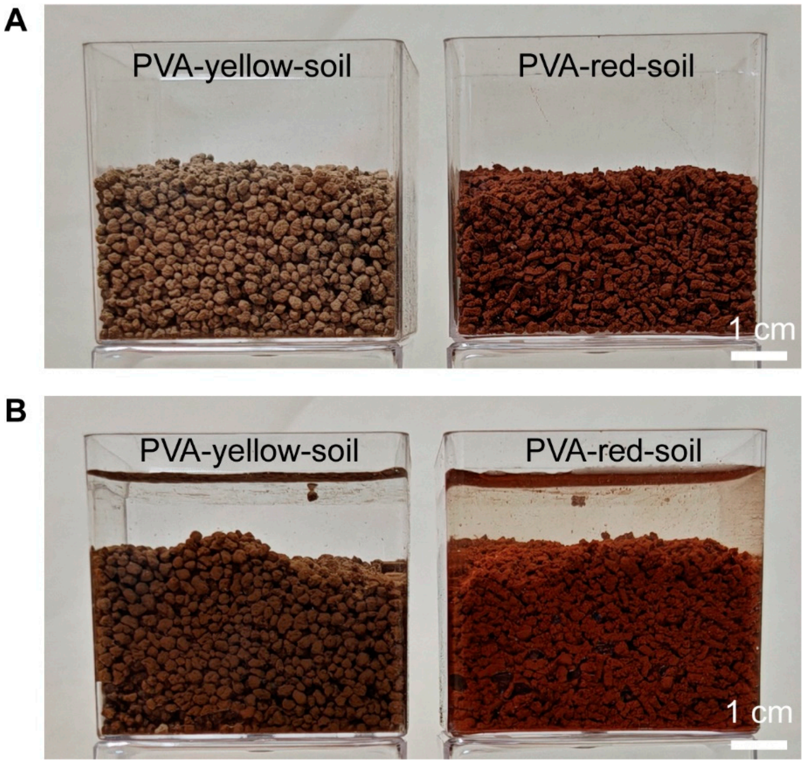

Figure S4. PVA-soil in dry state and wet state. Transferring from a dry environment (A) to an underwater condition does not break down the aggregate of PVA-soil (B).

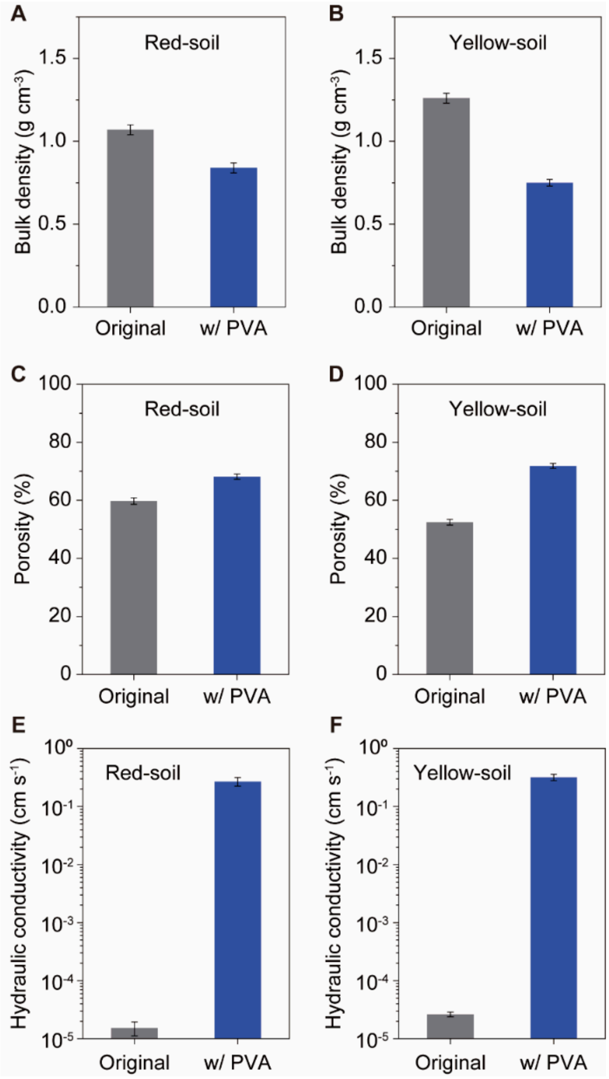

Figure S5. Comparison of physical properties of soil with and without the addition of PVA. A) Bulk density of red-soil. B) Bulk density of yellow-soil. C) Porosity of red-soil. D) Porosity of yellow-soil. E) Hydraulic conductivity of red-soil. F) Hydraulic conductivity of yellow-soil.

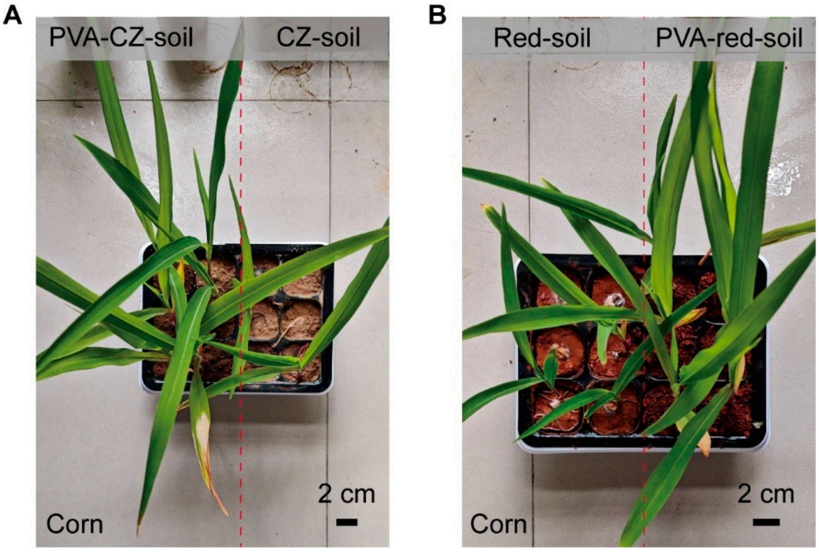

Figure S6. Using soil containing PVA and soil without PVA for corn cultivation. A) Corn seedlings grow well and all germinate in PVA-CZ-soil, but only 1/3 of the seedlings germinate and have weaker growth in CZ-soil. B) Corn seedlings grow well and all germinate in PVA-red-soil, but only half of the seedlings germinate in red-soil. Photos are recorded at day 14 after planting.

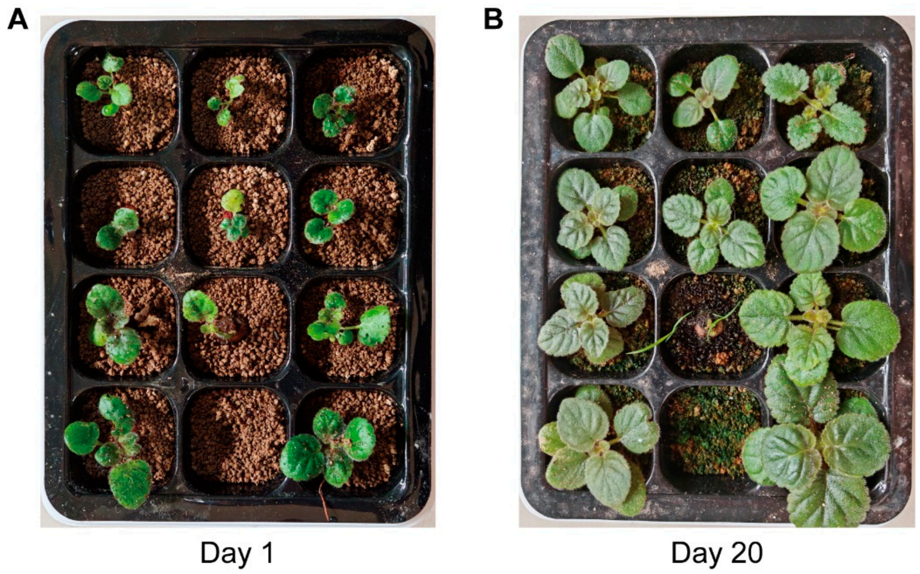

Figure S7. Growth of mini-cyclamen in PVA-yellow-soil. A) Mini-Cyclamen just transplanted into PVA-yellow-soil. B) Growth of Mini-Cyclamen after 20 days, with 10 out of 11 plants growing well.

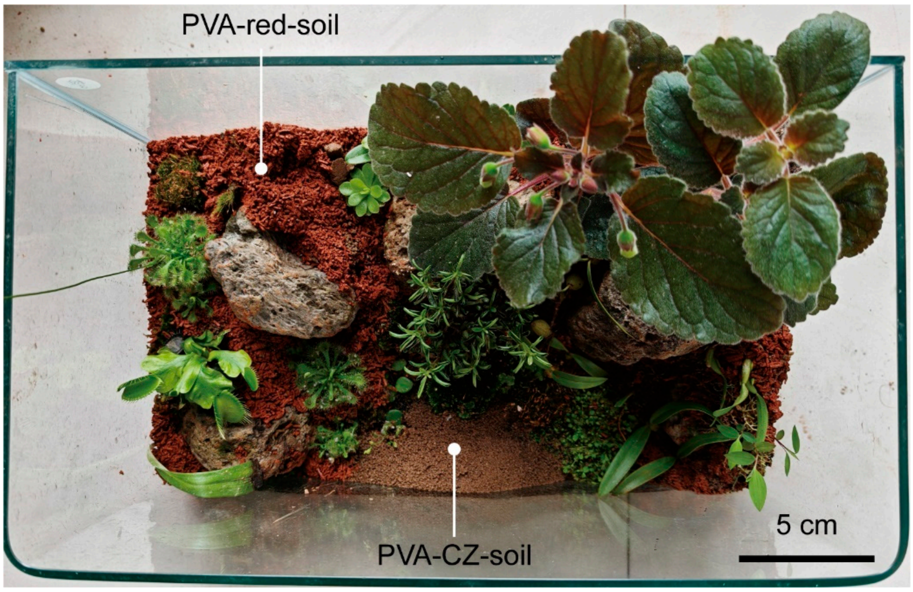

Figure S8. Plants grow in the PVA-red-soil and PVA-CZ-soil.

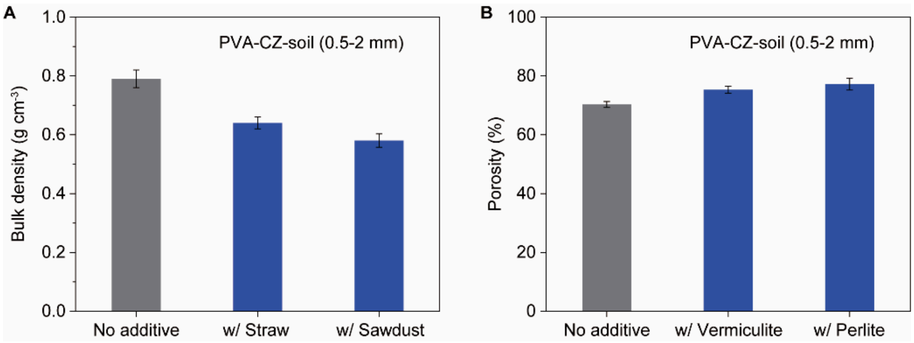

Figure S9. Comparison of physical properties of PVA-CZ-soil with and without the additive. A) The addition of 20 wt.% rice straw or sawdust reduces the bulk density of PVA-CZ-soil because rice straw and sawdust are much lighter than soil. In this experiment, straw and sawdust are approximately 2 mm in length. B) The inclusion of 10 vol% vermiculite and perlite increases the porosity of PVA-CZ-soil because these inorganic materials are porous. In this experiment, vermiculite and perlite are roughly 1 mm in size.

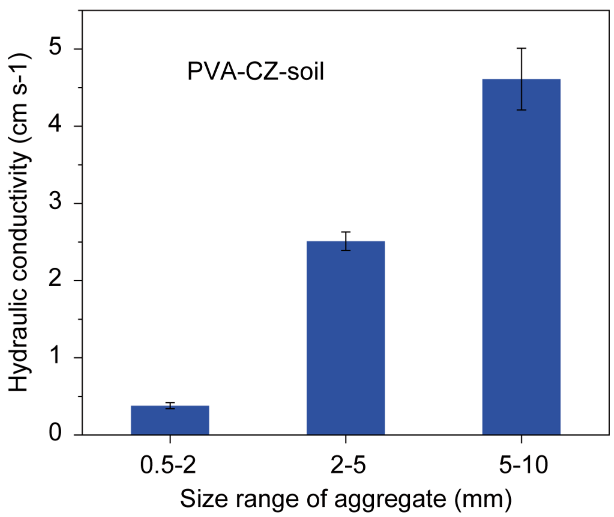

Figure S10. Hydraulic conductivity of PVA-CZ-soil with different aggregate sizes.
